# Fluorescence-based monitoring of electronic state and ion exchange kinetics with FCS and related techniques: from T-jump measurements to fluorescence fluctuations

**DOI:** 10.1007/s00249-017-1271-1

**Published:** 2017-12-19

**Authors:** Rudolf Rigler, Jerker Widengren

**Affiliations:** 1 Department of Medical Biochemistry and Biophysics, Karolinska Institute, Stockholm, Sweden; 2 Experimental Biomolecular Physics/ Department of Applied Physics, Royal Institute of Technology (KTH), Stockholm, Sweden

**Keywords:** T-jump, Fluctuations, Fluorescence correlation spectroscopy, Proton exchange, Photodynamics, Triplet state

## Abstract

In this review, we give a historical view of how our research in the development and use of fluorescence correlation spectroscopy (FCS) and related techniques has its roots and how it originally evolved from the pioneering work of Manfred Eigen, his colleagues, and coworkers. Work on temperature-jump (T-jump) experiments, conducted almost 50 years ago, led on to the development of the FCS technique. The pioneering work in the 1970s, introducing and demonstrating the concept for FCS, in turn formed the basis for the breakthrough use of FCS more than 15 years later. FCS can be used for monitoring reaction kinetics, based on fluctuations at thermodynamic equilibrium, rather than on relaxation measurements following perturbations. In this review, we more specifically discuss FCS measurements on photodynamic, electronic state transitions in fluorophore molecules, and on proton exchange dynamics in solution and on biomembranes. In the latter case, FCS measurements have proven capable of casting new light on the mechanisms of proton exchange at biological membranes, of central importance to bioenergetics and signal transduction. Finally, we describe the transient-state (TRAST) spectroscopy/imaging technique, sharing features with both relaxation (T-jump) and equilibrium fluctuation (FCS) techniques. TRAST is broadly applicable for cellular and molecular studies, and we briefly outline how TRAST can provide unique information from fluorophore blinking kinetics, reflecting e.g., cellular metabolism, rare molecular encounters, and molecular stoichiometries.

## T-jump and relaxation kinetics

In 1967, Manfred Eigen, together with Ronald G.W. Norrish and George Porter, were awarded the Nobel Prize in Chemistry, for their “studies of extremely fast chemical reactions, effected by disturbing the equilibrium by means of very short pulses of energy”. In the developed relaxation methods, chemical equilibria can be disturbed by intensive pulses, changing physical quantities like temperature, pressure, or electric fields. A variety of methods have been developed applying such pulses to observe the relaxation of a chemical equilibrium from one state into another one (Eigen and de Maeyer 1963). The Stockholm story in this field can be said to have begun when Rudolf Rigler came to Göttingen to work with Manfred Eigen on a temperature-jump (T-jump) apparatus. In this apparatus, fast heat generation could be accomplished by fast capacitor discharge, and subsequent observation of changes in light absorption compared to the equilibrium state was then available. In these absorption measurements, the difference in transmitted light was measured at very high light intensities in order to increase the signal-to-noise ratios with detectors allowing dynode switching (Rabl 1973).

With his experience in fluorescence spectroscopy Rigler (1969) started to develop a T-jump machine with fluorescence detection. In order to make fluorescence measurements a reality it was necessary to increase the total intensity of the emitted fluorescence. The construction of the fluorescence T-jump cell with the high aperture “fish eyes” was of key importance for providing adequate signal intensities. It was produced in the outstanding workshop of the Max-Planck Institute for Biophysical Chemistry, by the master mechanic Wolfgang Simm.

The first working fluorescence T-jump machine (Rigler et al. 1974) became quite popular and its reputation also attracted scientists outside of Göttingen. One day, Jean Pierre Changeux from the Institut Pasteur in Paris arrived with a bag of isolated nicotinic acetyl choline receptor, to be studied with the new fluorescence T-jump machine. However, the ensuing experiments took an unexpected end. Due to un-controlled conductivity in the solution of the receptor the electric pulse energy was dissipated fully into the measurement cavity and the valuable T-jump cell was destroyed. This moment of despair however led into new ideas for how to retrieve the information sought from the T-jump measurements. Thus this at the time unfortunate happening gave rise to the idea of using instead fluctuations in the equilibrium state, and coupled fluctuations in the optical signals, to follow the reaction. Together with Leo de Mayer and Klaus Gnädig, the first experiments were undertaken during 1968/1969, observing the association kinetics of acridin dyes and DNA for which data were available from T-jump measurements (Ramstein et al. 1980).

## Fluorescence correlation spectroscopy (FCS)

### Pioneer work in the 1970s

The first attempts to use fluorescence as the readout in fluctuation experiments illustrated that such analyses of systems under thermodynamic equilibrium can offer an attractive alternative to relaxation measurements, studying the response in a sample following some external perturbation. This general concept of fluctuation analysis was introduced already more than 100 years ago (Svedberg 1911; von Schmolukowski 1914; Chandrasekhar 1943) and over the years several techniques have been developed monitoring fluctuations in the number of particles or molecules of a specific type within a fixed sample volume. Well-known examples include the dynamic light scattering technique, exploiting the light scattering intensity from the particles of interest (Schaefer 1973; Berne and Pecora 1975), and the voltage clamp approach (Hodgkin et al. 1949), where fluctuation analysis of electrical currents over sections of cellular or artificial membranes is performed (with the later-developed patch clamp technique (Neher and Sakmann 1976) as its single-molecule counterpart).

Fluorescence correlation spectroscopy (FCS) thus belongs to an established category of fluctuation spectroscopy techniques, but with clear advantages coming with the use of the highly sensitive and specific fluorescence intensity signal as the fluctuating quantity. When Rudolf Rigler returned to the Karolinska Institute in 1970 to build up his own group, he received a letter from Elliot Elson, who learned relaxation kinetics from Robert Baldwin, a previous postdoc of Manfred Eigen in Göttingen. In this letter, Elson told Rigler about the results of their paper on FCS, which was published in 1972 (Magde et al. 1972). At that time, Ehrenberg and Rigler (1972) were involved in presenting for the first time a coherent description of the theory of rotational motion in the excited state using pulsed excitation. Ehrenberg and Rigler (1974) transposed this problem and its theory into the observation of fluctuations between the ground and excited state of fluorescent molecules, providing the description of excitation anti-bunching followed by rotational relaxation of the fluorescent species.

The theory and first experimental realizations of the FCS technique were thus in this way introduced during the years 1972–1974 (Magde et al. 1972, 1974; Ehrenberg and Rigler 1974; Elson and Magde 1974). Although showing great potential, the applicability of FCS was however strongly reduced at this time due to methodological constraints. In the measurements (Magde et al. 1972), the excitation beam was focused in a sample cell, and the fluorescence collected by a parabolic reflector. High background intensities, dominated by Raman scattering of the water molecules, and proportional to the size of the relatively large (> pL) detection volume, made it impossible to reduce the fluorophore concentrations without the background dominating over the fluorescence signal. As a consequence, the average number of fluorescent molecules in the detection volume had to be high, leading to lower relative fluorescence intensity fluctuations. Long measurement times were therefore required to distinguish and analyze these fluctuations, in turn imposing strict requirements on stability and absence of systematic noise in the optical and electronic parts of the instrumentation. The limited instrumental stability over long times, in combination with long time-range photochemical degradation, restricted the possible applications of FCS. Since FCS relies on the ability to detect and analyze fluctuations in the detected fluorescence signal stemming from dynamic events of single molecules, a major figure-of-merit for FCS measurements is the number of fluorescence photons that can be detected per molecule and time (the fluorescence brightness) (Koppel 1974). Consequently, high background light levels, low detection quantum yields, as well as the low capacity of the computers to analyze the fluctuation data, limited the applicability of FCS measurements at the time.

### Breakthrough in the 1990s

In the 1990s, more than 15 years after its first demonstration, the prerequisites for FCS measurements had greatly improved. Detection of individual fluorophore molecules, first in solid crystals under cryo-temperatures by absorption (Moerner and Kador 1989) and then by fluorescence (Orrit and Bernard 1990), led further to single-molecule detection in aqueous solution under room temperature (Shera et al. 1990). At the same time, the introduction of small, diffraction-limited observation volumes in FCS measurements, confocal epi-illumination, highly sensitive avalanche photodiodes for fluorescence detection and very selective band-pass filters to discriminate the fluorescence from the background, made it possible to improve signal-to-background ratios in FCS-measurements by several orders of magnitude (Rigler and Widengren 1990; Rigler et al. 1993). This development formed the basis for the FCS measurements as they are performed today.

**Fig. 1:**
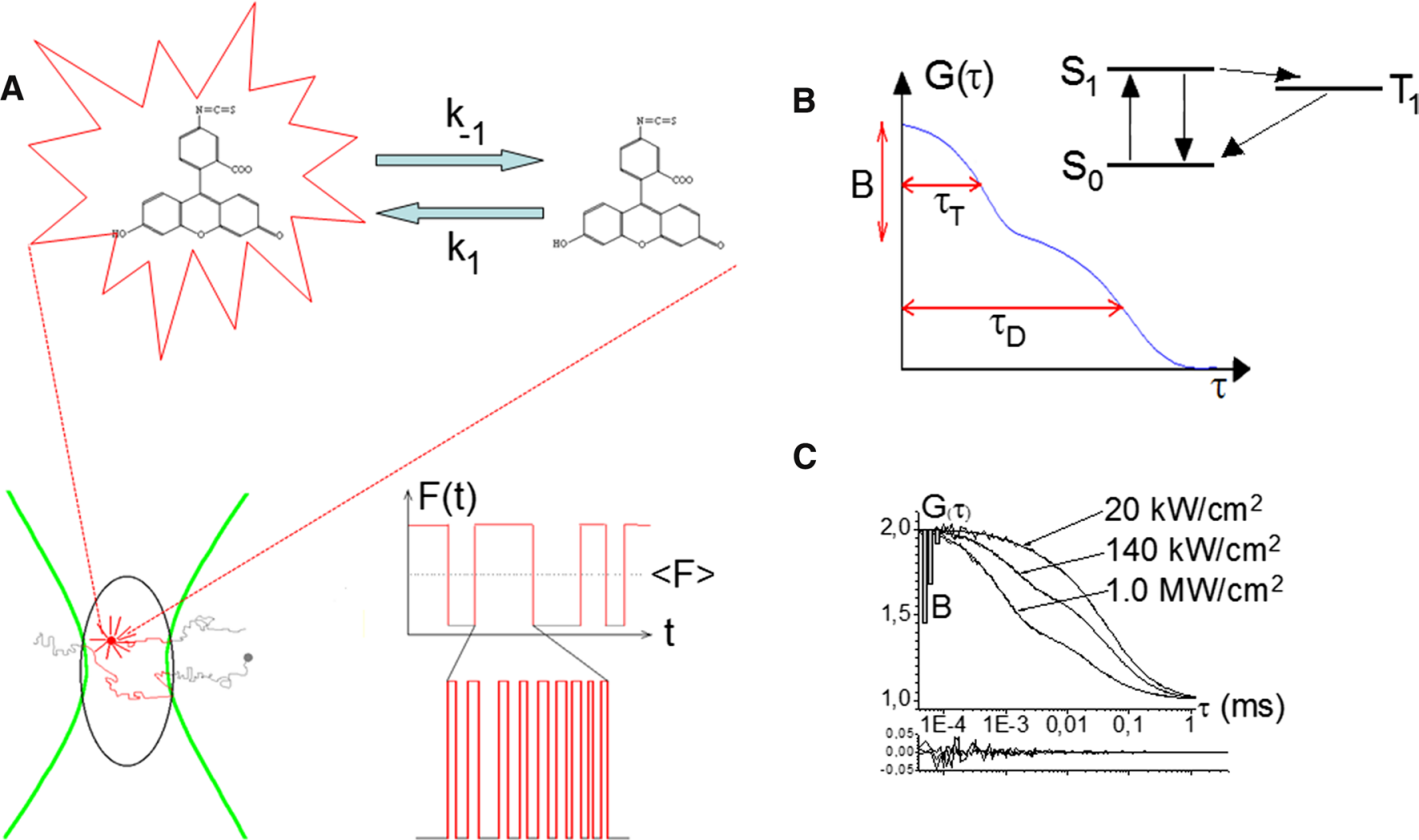
Monitoring of reaction kinetics at thermodynamic equilibrium by FCS. **a** Principal drawing of the type of reactions that can be studied. Fluorescent molecules undergoing Brownian diffusion through a confocal detection volume (*lower left*) also undergo a reaction, switching the fluorescence of the molecules on and off with rates *k*_1_ and *k*_−1_ (*upper part*). This reaction takes place on a faster time scale than the diffusion of the fluorescent molecules into and out of the detection volume. As a result, fluctuations in the fluorescence intensity, *F*(*t*), can be detected, with the faster time-scale fluctuations caused by the reaction superimposed on the slower fluctuations caused by diffusion (*lower right*). **b** Principal drawing of an FCS curve, recorded from fluorescent molecules undergoing fluorescence on–off blinking in the microsecond time-range, caused by transitions to and from the dark, long-lived, triplet state (*T*_1_). In the ACF of the recorded fluorescence intensity (Eq. 2), two relaxation processes will show up, as described by Eqs. 3 and 4. With the full amplitude of the ACF normalized to unity, the amplitude *B* corresponds to the average fraction of the fluorescent molecules in the detection volume which are in the triplet state. *τ*_T_ denotes the relaxation time for the singlet–triplet state transition and *τ*_D_ is related to the average dwell time of the fluorescent molecules in the detection volume. **c** Examples of FCS curves, recorded from the fluorophore Rh6G in water, with different excitation intensities, *I*_exc_, applied. Increasing *I*_exc_, leads to higher *B* and shorter *τ*_T_ values in the FCS curves. From the *I*_exc_-dependence of *B* and τ_T_, the transition rates to and from *T*_1_ can be determined

In an FCS measurement, in its most simple realization, fluorescence intensity fluctuations arise from translational diffusion, as the fluorescent molecules are diffusing into and out of a focused laser beam in an open confocal detection volume (Fig. 1a). For fluorescent molecules with a concentration, $c(\bar{r},t)$, the detected fluorescence intensity can then be written as (Rigler et al. 1993; Widengren et al. 1995):

1 $$F(t) = \varPhi_{\text{F}} \varPhi_{\text{D}} \iiint {{\text{CEF}}(\bar{r})k_{10} \frac{{\sigma I_{\text{exc}} (\bar{r})}}{{\sigma I_{\text{exc}} (\bar{r}) + k_{10} }}c(\bar{r},t){\text{d}}V = }\varPhi_{\text{F}} \varPhi_{\text{D}} \iiint {W(\bar{r})c(\bar{r},t){\text{d}}V}.$$

Here, *Ф*_F_, *σ* and *k*_10_ denote the fluorescence quantum yield, excitation cross section, and deactivation rate of the fluorescent molecules. *Ф*_D_ and ${\text{CEF}}(\bar{r})$ signify the detection quantum yield and the collection efficiency function of the instrument. $I_{\text{exc}} (\bar{r})$ denotes the excitation intensity of the laser. $W(\bar{r})$ is the molecular detection efficiency.

From the fluctuations in *F*(*t*), denoted *δF*(*t*), information can be retrieved about the translational diffusion coefficients, *D*, and the average number of molecules, *N*, residing simultaneously in the detection volume. Assuming that $W(\bar{r})$ has a Gaussian distribution is both the radial and axial dimensions, and in the absence of any other kinetic process than translational diffusion affecting the fluorescent molecules, the time-dependent normalized intensity autocorrelation function (ACF) can be written (Magde et al. 1972, 1974):

2 $$\begin{aligned} G(\tau ) & = \frac{ < F(t) F(t + \tau ) > }{{ < F >^{2} }} = \frac{{ < \left[ { < F > + \delta F(t)} \right]\left[ { < F > + \delta F(t + \tau )} \right] > }}{{ < F >^{2} }} \\ &= \left\{ {F(t){\text{ is stationary}}} \right\} = \frac{ < \delta F(t)\delta F(t + \tau ) > }{{ < F >^{2} }} + 1 = \frac{1}{N}\left( {\frac{1}{{1 + {{4D\tau } \mathord{\left/ {\vphantom {{4D\tau } {\omega_{1}^{2} }}} \right. \kern-0pt} {\omega_{1}^{2} }}}}} \right)\left( {\frac{1}{{1 + {{4D\tau } \mathord{\left/ {\vphantom {{4D\tau } {\omega_{2}^{2} }}} \right. \kern-0pt} {\omega_{2}^{2} }}}}} \right)^{{{\raise0.7ex\hbox{$1$} \!\mathord{\left/ {\vphantom {1 2}}\right.\kern-0pt} \!\lower0.7ex\hbox{$2$}}}} + 1. \\ \end{aligned}$$

*G*(*τ*) gives a measure of how the fluorescence intensity detected at a certain time, *F*(*t*), is related to that detected at a correlation time, *τ*, later, i.e., to *F*(*t* + *τ*). In Eq. 2, brackets denote average over the measurement time, which should be long enough to secure convergence of *G*(*τ*). *ω*_1_ and *ω*_2_ signify the distances from the center of the detection volume in the radial and axial dimensions, respectively, at which $W(\bar{r})$ has decreased by a factor of e^2^. With knowledge about *ω*_1_ and *ω*_2_, e.g., from FCS calibration measurements with fluorophores with known diffusion coefficients, *D* for the fluorescent species studied can be determined. Equation 2 is based on the assumption that *F*(*t*) can be considered a stationary and ergodic process (essentially that its average and variance is constant over time, and that the average of *F*(*t*) from one or a few fluorescent molecules recorded over a longer time should be the same as an instant average of very many fluorescent molecules). Further assumptions are that the FCS measurement is performed at equilibrium in an ideal solution, with no photobleaching and in a sample volume ≫ than the confocal detection volume.

### FCS for monitoring of reaction kinetics

FCS is not limited to analyses of the number and diffusion properties of fluorescent molecules. Even for a standard FCS instrument with a single-point, stationary detection volume, a wide range of processes can be studied, spanning a time range from sub-nanoseconds to seconds. In principle, any process at equilibrium conditions, which reflects itself as a change of the detected fluorescence *F*(*t*), can be measured, given that it occurs within the dwell time of the fluorescent molecules in the detection volume ($\tau_{\text{D}} \approx \omega_{1}^{2} /4D$). Correlation analyses can also be performed on fluorescence intensities recorded in e.g., different spectral channels [fluorescence cross-correlation spectroscopy, FCCS (Ricka and Binkert 1989; Schwille et al. 1997)], spatial locations [image correlation spectroscopy, ICS (Brinkmeier et al. 1999; Petersen et al. 1993; Digman et al. 2005)], or based on fluorescence lifetime changes [fluorescence lifetime correlation spectroscopy, FLCS (Benda et al. 2006)]. It is beyond the scope of this review to discuss the full range of molecular dynamic processes that can be studied by FCS. In this review, we will rather discuss how FCS can be used to study reaction kinetics at thermodynamic equilibrium, as an alternative to relaxation experiments, for reactions which generate changes in the fluorescence brightness, *Q*, of the molecules. In its easiest realization, as depicted in Fig. 1a, the (reversible) reaction to be studied involves one fluorescent species, takes place on a time scale much faster than the passage times of the molecules through the detection volume, and switches the fluorescence completely on and off, yielding high contrast fluorescence intensity fluctuations, superimposed on those due to translational diffusion of the molecules in and out of the detection volume. The ACF recorded from such a sample (Fig. 1b) can then be separated into two factors. The first factor, *G*_D_(τ), depends on the transport properties (diffusion or flow) of the molecules and the second, *R*(τ), depends only on the reaction rate constants (Palmer and Thompson 1987):

3 $$G(\tau ) = G_{\text{D}} (\tau )R(\tau ) + 1.$$

In this particular case, the separate reaction-related factor can be written as:

4 $$R(\tau ) = \frac{1}{1 - B}\left( {1 - B + B\exp ( - k_{\text{B}} \tau )} \right)$$

where *B* denotes the average fraction of the fluorescent molecules in the detection volume which are in the dark state. $k_{\text{B}} = 1/\tau_{\text{B}}$ is the relaxation rate of the reaction, and is given by the sum of the fluorescence on and off rates, *k*_1_ and *k*_−1_. In a more general case, the separation of *G*(*τ*) into two factors as in Eq. 3 is possible for reactions in which the diffusion of the reactants and product molecules is much slower than the chemical relaxation time(s) and/or the diffusion coefficients of all fluorescent species can be considered equal (Palmer and Thompson 1987). For a fluorescent species studied by FCS, and undergoing chemical reactions, the second factor can more generally be expressed as:

5 $$R(\tau ) = \frac{{\sum\nolimits_{i,j = 1}^{M} {Q_{i} Q_{j} X_{ij} (\tau )} }}{{\sum\nolimits_{i = 1}^{M} {Q_{i}^{2} \bar{C}_{i} } }}.$$

Here, *Q*_*i*_ is the fluorescence brightness coefficient of state *i* and *X*_*ij*_(*τ*) is the solution to the following set of differential equations and initial conditions:

6 $$\begin{aligned} {\text{d}}X_{ik} (\tau )/{\text{d}}\tau &= \sum\limits_{j = 1}^{M} {T_{ij} X_{jk} (\tau )} \\ X_{ik} (0) &= \bar{C}_{i} \delta_{ik} , \\ \end{aligned}$$

where *δ*_*ik*_ = 1 if *i* = *k*, and *δ*_ik_ = 0 if *i* ≠ *k*. *X*_*ij*_(*τ*) describes the probability of finding a molecule in state *j* at time *τ*, given that it was in state *i* at time 0. *M* is the number of species participating in the chemical reaction, and *T*_*ij*_ represents the corresponding matrix of the kinetic rate coefficients. In Eq. 5, it can be noted that in FCS measurements, each different species analyzed is weighted by the square of its fluorescence brightness. For an ACF recorded from a sample containing several different fluorescent species, it can therefore be strongly misleading to interpret the inverse amplitude of the ACF as the true average number of fluorescent molecules (1/*G*(0) = *N* in Eq. 2). More generally, for *M* different species, with brightnesses *Q*_*i*_ and average numbers *N*_*i*_ (and disregarding fluorescence anti-bunching) (Magde et al. 1974; Widengren and Mets 2001):

7 $$1/G(0) = \frac{{\mathop \sum \nolimits_{i = 1}^{M} \left[ {N_{i} Q_{i} } \right]^{2} }}{{\mathop \sum \nolimits_{i = 1}^{M} N_{i} Q_{i}^{2} }}.$$

When applicable, it is very convenient to treat the kinetics of a chemical reaction separately from the translational diffusion in the fluctuation analysis, as given by Eq. 2. This treatment applies to a rather broad range of chemical reactions such as inter- or intra-molecular dynamics, influenced by fluorescence quenching (Bonnet et al. 1998; Chattopadhyay et al. 2002). Moreover, for a reaction that under certain conditions does not fulfill the criteria, it is sometimes possible to modify the conditions. For instance, the dwell times can be retarded with respect to the chemical relaxation times by expanding the detection volume, or the reactions under study can be speeded up, e.g., by using higher concentrations of un-labelled reactants (Widengren et al. 1995, 1999, 2007; Widengren and Rigler 1997).

In the sections below, we will discuss two realizations of this FCS approach to monitor reaction kinetics, generating changes in *Q* of the fluorescent species studied. First, it will be shown how FCS can be used to monitor a range of photo-induced transitions in fluorophores. Second, how monitoring of ion-sensitive fluorophores by FCS offers an alternative way of monitoring proton exchange kinetics and how this approach can be used to investigate protonation kinetics at biological membranes.

### FCS for photodynamic characterization of fluorescent species

Photophysical properties of the fluorescent molecules under study set the fundamental limits for the overall performance of virtually all forms of fluorescence spectroscopy and imaging, where high sensitivities, read-out rates and/or resolutions are required. Similarly, these properties also set the ultimate limits for FCS measurements. Population of photo-induced dark states, such as triplet, photo-isomerized, and photo-oxidized states reduce the fluorescence brightness of the fluorophore molecules studied, a major figure-of-merit for FCS measurements (Koppel 1974). Some of these states may also act as precursor states for photobleaching, and the blinking caused by these transitions may cause problems in FCS and in single-molecule experiments, in that they may shadow other molecular processes of interest, taking place in the same time range. On the other side, based on the general approach to study reaction kinetics via changes in the fluorescence brightness *Q* (Eqs. 3–7), FCS has also turned out to be a very suitable tool to study these transitions. Figure 1c shows FCS curves recorded from the fluorophore rhodamine 6G (Rh6G) in air-saturated aqueous solution, and how the average population of the dark, lowest triplet state of Rh6G, given by the relative amplitude B, as well as the singlet–triplet state relaxation time, *τ*_T_, corresponding to *τ*_B_ in Eq. 4, vary with the excitation irradiance within the confocal detection volume in the FCS experiment. From the excitation irradiance dependence observed in the FCS curves (a so-called FCS power series), the transition rate constants to and from *T*_1_ can be determined in a straightforward manner (Widengren et al. 1995). Similarly, a whole range of photo-induced dark transient states can be kinetically characterized, including photo-ionized (Widengren et al. 1997) and photo-isomerized states (Widengren and Schwille 2000; Widengren and Seidel 2000), as well as the influence of chemical additives and environmental conditions on these transitions (Widengren et al. 1995, 1997, 2007; Widengren and Schwille 2000). Likewise, the overall photostabilities of fluorophores under excitation conditions required for FCS and other forms of ultrasensitive fluorescence spectroscopy and imaging can be studied (Widengren et al. 2007; Eggeling et al. 1998; van den Berg et al. 2001). Interestingly, in such studies, compounds known in the fluorescence spectroscopy field as fluorescence quenchers, such as potassium iodide, may under excitation conditions for FCS and single-molecule fluorescence spectroscopy turn out to act as anti-fading compounds (Chmyrov et al. 2010). Similar transitions as in organic fluorophore molecules can also be found in green fluorescent proteins (GFPs). In GFPs, the transitions are however far less influenced by environmental parameters, since the fluorescently active unit is located in the inner part of the GFPs, shielded from the surroundings by a tight beta sheet barrel structure (Widengren et al. 1999; Haupts et al. 1998).

Compared to e.g., transient-state absorption/flash photolysis (Van Amerongen and Van Grondelle 1995; Korobov and Chibisov 1978) and phosphorescence studies (Jovin and Vaz 1989) the FCS approach offers some advantages. For triplet state studies, it uses the highly sensitive fluorescence readout to monitor the triplet state, rather than the faint, easily quenched, phosphorescence signal from the triplet state itself. Thereby, a favorable combination of a high signal level (given by the readout of fluorescence photons) and an outstanding environmental sensitivity (given by the long lifetimes of the transient states) can be obtained. Quenching of the triplet states of the fluorophores by oxygen or other compounds will not ruin the read-out signal. Compared to flash photolysis the experimental realization is relatively simple and more easily applicable to a broader range of samples.

### FCS for studies of proton exchange dynamics in solution and on biomembranes

**Fig. 2:**
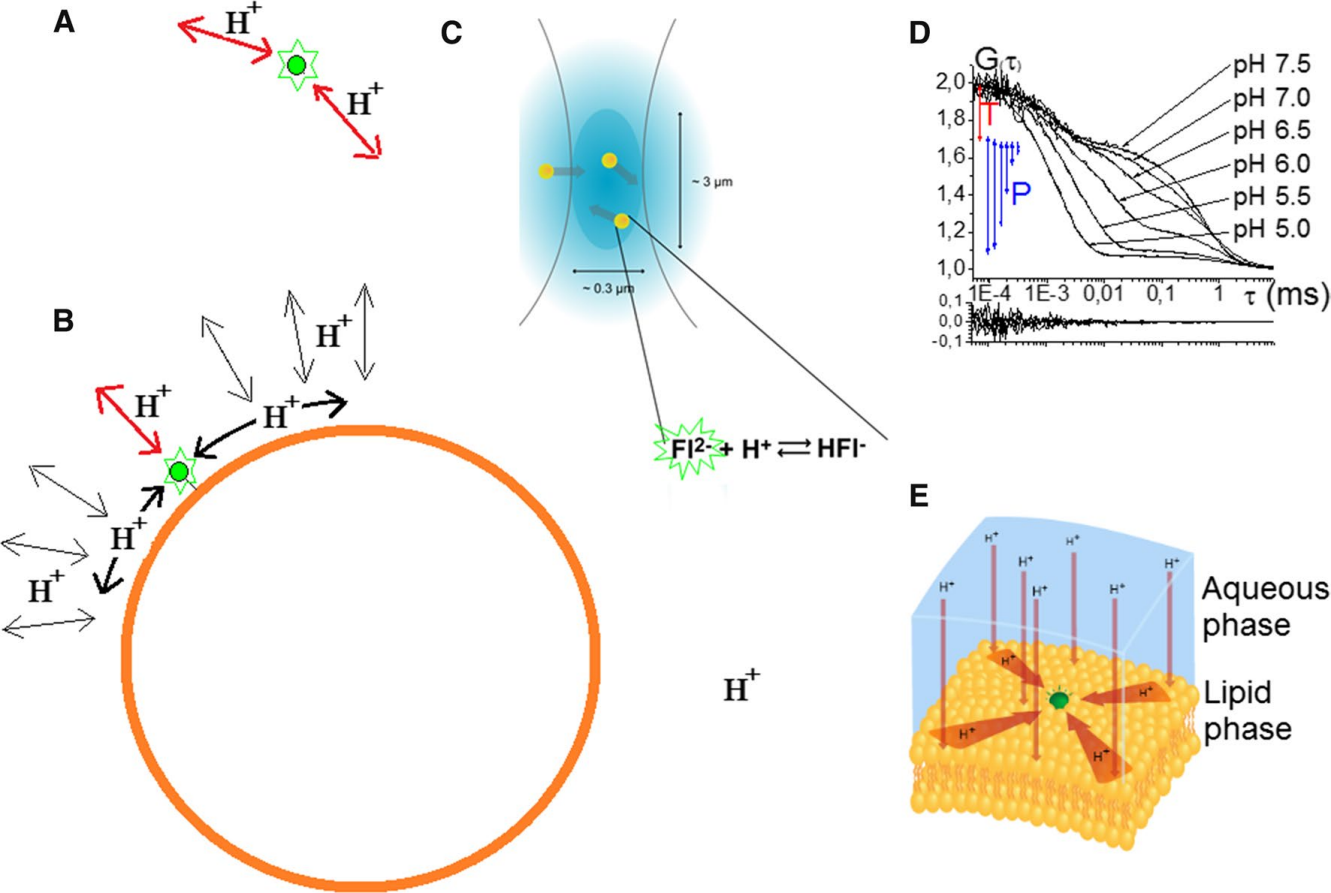
Protonation kinetics in solution and at biological membranes, studied by FCS. Proton exchange to and from pH-sensitive fluorophores results in fluorescence blinking, which can be analyzed by FCS as outlined in Fig. 1, and by use of Eq. 4. For fluorophores free in solution (**a**), FCS measurements show that the protonation on-rate is two orders of magnitude slower than when the fluorophores are located close to a lipid membrane of a small unilamellar vesicle (SUV) (**b**). Apart from direct exchange of protons between the fluorophore and the bulk (red arrows), the membrane–water interface also exchange protons with the bulk (*thin black arrows*), and protons at the surface may migrate along the surface (*thick black arrows*) and reach the fluorophore, before dissociating from the membrane surface into the bulk. **c** The proton exchange to and from fluorophores, located at the membranes of SUVs, were measured on SUVs freely diffusing into and out of the detection volume of the FCS instrument. **d** Examples of FCS curves recorded from the pH-sensitive fluorophore Fluorescein in water at different pH. Three relaxations can be observed in the FCS curves: singlet–triplet state relaxation in the µs time range (with amplitude T, marked red, reflecting the fraction of fluorophores being in the triplet state while passing the detection volume), proton exchange in a time range of 1–100 µs (with the amplitude P, marked blue, related to the fraction of fluorophores in the protonated, close-to non-fluorescent state of Fluorescein), and diffusion relaxation beyond 100 µs (with the detection volume expanded well beyond the diffraction limit in this series of measurements). **e** Illustration of a membrane acting as a proton collecting antenna, having a radius given by how far a proton can diffuse along the surface before the probability to find it on the surface equals the average value on the surface

Monitoring blinking rates and the fractions of fluorescent and non-fluorescent fluorophores by FCS, as outlined above (Eqs. 3–7), can also be applied to characterize ion exchange to and from ion-sensitive fluorophores at thermodynamic equilibrium (Widengren and Rigler 1997; Widengren et al. 1999). In such measurements, taking as an example a pH-sensitive dye in a buffered aqueous solution, which is non-fluorescent in its protonated form, the recorded FCS curves can be described by Eqs. 3 and 4 (Fig. 1b). The amplitude *B* then corresponds to the fraction of non-fluorescent protonated fluorophores, and the relaxation rate *k*_B_ to the sum of the protonation and de-protonation rates of the fluorophores (Fig. 2a, c). With knowledge of the p*K*_a_ (and $K_{\text{a}} = 10^{{ - {\text{p}}K_{\text{a}} }}$) of the fluorophore, the local pH (and $\left[ {H^{ + } } \right] = 10^{{ - {\text{pH}}}}$) can then be determined from the relaxation amplitude B in the ACFs (Eq. 4). If the fluorophore becomes non-fluorescent upon protonation (Widengren and Rigler 1997; Widengren et al. 1999):

8a $$B = \frac{{\left[ {H^{ + } } \right]}}{{\left[ {H^{ + } } \right] + K_{\text{a}} }},$$

if it is fluorescent in the protonated form and becomes non-fluorescent upon de-protonation:

8b $$B = \frac{{K_{\text{a}} }}{{\left[ {H^{ + } } \right] + K_{\text{a}} }}.$$

Moreover, in a buffered aqueous solution, *k*_B_ directly reflects and depends linearly on the local buffer concentration (Widengren et al. 1999).

Dyes used for FCS studies of proton (or ion exchange) do not have to be completely non-fluorescent upon protonation (or de-protonation). However, the relaxation amplitude *B* will get smaller the less distinct the brightness difference upon protonation is. Introducing *Q* as the relative brightness of the dimmer form of the dye, protonated or de-protonated, the amplitudes in Eqs. 8a and 8b will change into (Widengren and Schwille 2000):

9 $$B_{\text{Q}} = \frac{{B(1 - Q)^{2} }}{{1 + Q^{2} (1 - B)}}.$$

Naturally, if there is no difference in brightness of the fluorophore upon protonation (*Q * = 1), *B*_Q_ will be zero and no relaxation can be observed in the ACFs. For many pH-sensitive fluorophores *Q* is small (1–2%), and then only marginally affects the relaxation amplitudes. With knowledge of *Q* it can also be properly corrected for. Alternatively, higher-order correlation analyses of *δF*(*t*) can be applied to resolve *Q*, as recently demonstrated (Abdollah-Nia et al. 2017). As an additional alternative, ratio-metric pH-sensitive dyes can be used, for which the excitation and/or the emission spectrum changes upon protonation. For such dyes, two or several *Q* values can be included, effective for different laser excitation wavelengths and/or detection within different wavelength bands. If the excitation/emission in one wavelength band increases upon protonation of the dye, it normally decreases in another wavelength band. FCCS measurements, recording the cross-correlation of intensities recorded at different excitation and/or emission wavelengths, then typically display negative *B*_Q_ relaxation amplitudes (Persson et al. 2009).

The FCS-based approach for ion exchange studies can offer selective advantages over other techniques for measuring local ion concentrations, and in particular exchange kinetics of ions on a local scale. We have exploited these advantages in a series of papers to study proton exchange at biological membranes (Brändén et al. 2006; Öjemyr et al. 2009; Sanden et al. 2010; Xu et al. 2016; Sjöholm et al. 2017). Proton gradients across biological membranes act as driving forces for many energy-consuming cellular processes, not the least ATP synthesis by ATP synthase in the mitochondria. To generate the gradients, proton transport at and across membranes is required and involves a series of membrane-spanning proteins in the inner membranes of the mitochondria. The underlying mechanisms for this proton transport has been subject to extensive research (Medvedev and Stuchebrukhov 2011), but is nonetheless not completely understood. One of the key questions concerns the nature of coupling between proton generators, such as cytochrome *C* oxidase (Cyt*c*O), pumping protons across the membrane, and proton consumers, such as ATP synthase, using the proton gradients across the membrane to drive the ATP synthesis (Medvedev and Stuchebrukhov 2011). Both the outlet of the generator and inlet of the consumer proteins are located on the same side of the membrane, but the proteins are spatially separated. A major question is how the generated proteins get to the consumers before they are dissociated from the membrane surface. A major experimental technique for these molecular proton exchange studies is the laser-induced proton pulse approach (Gutman 1986) and other relaxation techniques. Basically, a light flash directed to one side of a membrane releases protons from caged compounds in the membrane, and the response from pH-sensitive fluorophores is then monitored on the other side of the membrane. The FCS approach above can offer complementary points of view on these relaxation techniques. In particular, in FCS measurements protons associating to and dissociating from pH-sensitive fluorophores are observed at equilibrium conditions. No perturbation into some, often strongly un-physiological, initial condition is required. Moreover, while protonation kinetics measurements by proton pulse techniques require 1–5% of the lipids in the membranes to be labeled (Serowy et al. 2003), FCS is typically performed at about 1000-fold lower concentrations. Thereby, the influence of buffering effects due to ions binding to the fluorophores themselves, or to any light absorbing proton emitter, can be avoided. Finally, the proton exchange seen in the FCS measurements reflects the conditions in the immediate surroundings of individual fluorescent probes, in contrast to proton pulse measurements (Serowy et al. 2003), where the measured protonation dynamics reflect the conditions in the media the protons pass on their way from light absorbing proton emitters to the pH-sensitive fluorophores.

Based on these selective advantages of the FCS approach we have investigated the principal role of biological membranes for proton uptake of membrane incorporated proteins (Fig. 2a–e). Interestingly, we found that the protonation rate of the pH-sensitive dye fluorescein increased by two orders of magnitude (from ~ 10^11^ to ~ 10^13^ M^−1^ s^−1^) when labeled to a lipid in a membrane of a small unilamellar vesicle (SUV, Fig. 2b), compared to when free in solution (Fig. 2a) (Brändén et al. 2006). In comparison, FCS measurements in membranes with negatively charged head-groups instead of zwitterionic head-groups only changed the protonation rate by a factor of two (Brändén et al. 2006). This finding gives direct evidence that the membrane-water interface can act as a proton collecting antennae (PCA), with an area far exceeding the size (or the physical cross section for protonation) of the fluorophore itself. In follow-up studies, the same enhancement of more than two orders of magnitude in the protonation on-rate was also found when the fluorophore label was placed on the proton generator Cyt*c*O, when free in solution, compared to when in the membrane of an SUV (Öjemyr et al. 2009). The PCA effect is less observable at lower pH (< 7), when the proton association rate via the membrane becomes comparable to that of direct protonation of the fluorophore from the bulk solution (Fig. 2, left bottom) (Sanden et al. 2010). The radius of the PCA, R_PCA_, is related to how far a proton generated at the membrane surface can diffuse from its site of generation before the probability to find a proton equals the average value on the surface (Gutman and Nachliel 1995). From proton pulse experiments, the R_PCA_ of lipid membranes has been determined to be as large as tens of micrometers, and the diffusion coefficients of the protons, D_S_, along the membrane-water interfaces as high as 5 × 10^−5^ cm^2^/s (Medvedev and Stuchebrukhov 2011; Serowy et al. 2003). In contrast, from FCS experiments D_S_ is two orders of magnitude lower (Brändén et al. 2006), and protonation dynamics measurements by FCS on lipid nanodiscs, with diameters around 10 nm, show that the protonation on-rate of fluorescein remains as high as in SUVs (diameter of ~ 30 to 100 nm), even when incorporated into the small membrane areas of the nanodiscs. A reasonable interpretation is that the density of protonatable groups bound to the membrane surface can sustain a higher local proton concentration at the surface than in the bulk solution, and a higher proton exchange rate of a surface group (Fluorescein) with the surface than with the bulk solution (even if the diffusion coefficient of protons in bulk water is considerably higher than along the membrane) (Brändén et al. 2006). The dependence of the protonation on-rate on the buffer concentration in the bulk water and Monte-Carlo simulations of the proton exchange to and from a fluorophore at a nanodisc of different size further confirmed that both R_PCA_ and D_S_ along lipid membranes are 2–3 orders of magnitude smaller, when measured by FCS (Xu et al. 2016), as compared to proton pulse data (Medvedev and Stuchebrukhov 2011). The underlying reason for this quite significant difference can likely be attributed to the fact that FCS measures the very local proton exchange kinetics, in the very surroundings of individual fluorophores (nanometer scale), while proton pulse measurements monitor the propagation of proton pulses over distances beyond millimeters. The longer distance measurements capture an average migration behavior of the protons, including “hopping” between the lipid head group region of the membrane, the membrane-water interface and the bulk water. In contrast, FCS measurements capture the local exchange dynamics at the lipid head group region and membrane-water interface only. These results thus emphasizes the ability of FCS to monitor the proton exchange at the very local scale, and also provide experimental support to recent theoretical studies showing that proton diffusion along membrane surfaces is time- and length-scale dependent (Wolf et al. 2014; Yamashita and Voth 2010).

## Transient-state (TRAST) spectroscopy/imaging to exploit the information content of fluorescence blinking kinetics

### Concept for TRAST

**Fig. 3:**
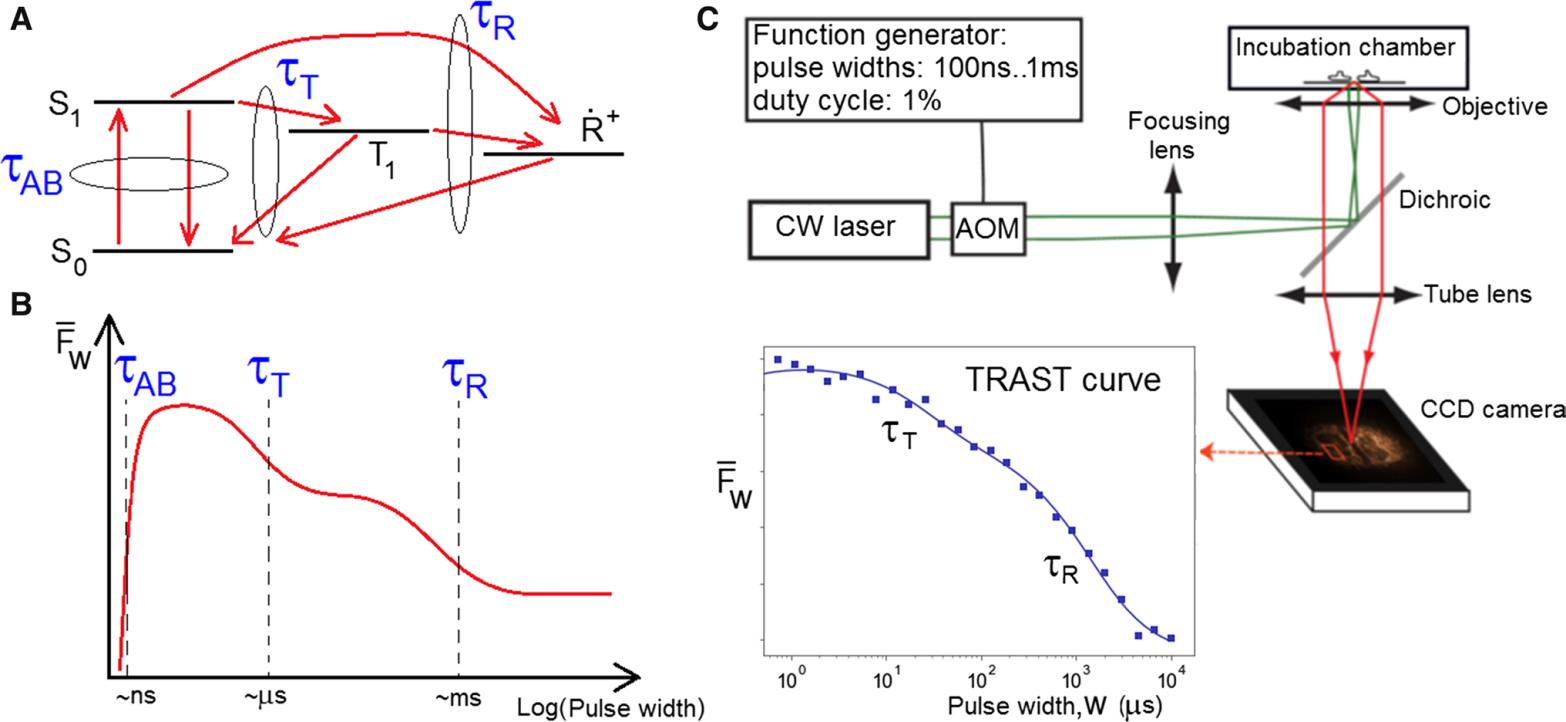
Illustration of how fluorophore photodynamics is monitored in transient state (TRAST) spectroscopy/imaging. **a** Electronic state model for a fluorophore, comprising the ground and excited singlet states (*S*_0_ and *S*_1_), the lowest triplet state (*T*_1_) and a photo-oxidized state ($\dot{R}^{ + }$). Following onset of constant excitation, *τ*_AB_, *τ*_T_ and *τ*_R_ denote the relaxation times for the population of the singlet states, *T*_1_ and $\dot{R}^{ + }$, respectively. **b** For the same fluorophore, subject to rectangular excitation pulses, the graph illustrates how the average fluorescence, $\bar{F}_{\text{W}}$, can vary with the duration, *w*, of the excitation pulses. This so-called TRAST curve reflects the population build-up of the electronic states in (**a**) upon onset of excitation, and the relaxation times *τ*_AB_, *τ*_T_ and *τ*_R_. **c** Principle setup for TRAST imaging. The beam of a continuous wave (CW) laser is passed through an acousto-optical modulator, generating rectangular excitation pulse trains with varying pulse durations, *w*. These are fed into a microscope and via its objective onto the sample. The fluorescence generated by the excitation pulse trains is collected by the same objective and recorded by a camera in the image plane. By recording images of the average fluorescence, $\bar{F}_{\text{W}}$, for different duration, *w*, of the excitation pulses, TRAST curves can be generated within regions of interest in the image, and TRAST images can be obtained, mapping e.g., *τ*_T_ and *τ*_R_, or the underlying transition rates to and from *T*_1_ and $\dot{R}^{ + }$

As discussed above, fluorophore blinking, caused by population dynamics of photo-induced, long-lived, non-fluorescent triplet (*T*_1_), photo-oxidized ($\dot{R}$ ^+^), photo-reduced ($\dot{R}$ ^−^) or photo-isomerized states (Fig. 3a) is of central importance to all forms of fluorescence-based ultrasensitive and ultrahigh-resolution spectroscopy/imaging. While blinking has to be suppressed in single-molecule fluorescence studies (reduces brightness and obscures observation of other dynamic processes), blinking is an absolute prerequisite for all forms of super-resolution microscopy [see e.g., (Eggeling et al. 2015) and (Blom and Widengren 2017) for reviews]. Moreover, the long lifetimes of these non-fluorescent states, ~ 10^−6^ to 10^−3^ s, compared to ~ 10^−9^ s for the fluorescence lifetime of the excited singlet state, make these states highly environment sensitive. These states thus represent a whole set of additional parameters, which in a very sensitive manner can reflect microenvironments as well as biomolecular dynamics and interactions of fluorescent molecules (Widengren 2010). This possibility seems not to have been fully realized in the fluorescence field until more recently (Weidemann et al. 2014; Kawai et al. 2015; Mahoney et al. 2015; Bag and Wohland 2014; Querard et al. 2015).

FCS-based monitoring of these states combines high detection sensitivity by the fluorescence readout, with high environmental sensitivity, given by the long lifetimes of the transient states. However, FCS measurements require fluorescent molecules with high brightness and in low concentrations, and highly sensitive detectors with high time resolution. Although FCS provides a very powerful and versatile means to analyze photodynamic events, this limits the application range and throughput of FCS measurements, and makes parallel readouts and imaging difficult.

To overcome these limitations, we have introduced an approach, so-called transient-state (TRAST) imaging (Sanden et al. 2007, 2008), where the fluorescent sample is subject to a modulated excitation (Fig. 3). Depending on the excitation pulse train characteristics (e.g., pulse duration, separation and height) long-lived photo-induced transient states (e.g., triplet, photo-isomerized and photo-ionized states) of the fluorophores in the sample will be populated to different extents. Figures 3a, b illustrate how the fluorescence intensity varies with time after onset of excitation, and how equilibration between the ground and the excited singlet states (*S*_0_ and *S*_1_) typically takes place within nanoseconds after onset of excitation, between the singlet and triplet (*T*_1_) states within microseconds, and finally how a population of photo-oxidized (or –reduced) states ($\dot{R}^{ + } )$ build up and equilibrate within milliseconds after onset of excitation. Upon transient-state population build-up in the sample, the fluorescence intensity will drop in a corresponding manner. By systematically varying the excitation pulse train characteristics, e.g., by use of an acousto-optical modulator (AOM), and registering how the plain time-averaged fluorescence intensity changes with e.g., the pulse width (a so-called TRAST curve, Fig. 3c) the population kinetics of the fluorophore transient states can then be retrieved. As with FCS, the TRAST technique combines the high detection sensitivity of the fluorescence readout with the high environmental sensitivity of the dark transient states, but does not rely on a high fluorescent brightness of the molecules studied, on a high time resolution, or on a certain concentration of the sample. This makes TRAST imaging a broadly applicable approach, applicable e.g., on live cells, and allows transient states to be imaged by e.g., a standard CCD camera. For live cell studies by light microscopy in general, photo-toxic and photo-sensitizing effects from both endogenous and exogenous (fluorophore) compounds have to be considered. These effects depend on several parameters, such as the concentration of these compounds in the cells, the wavelength of the irradiating light, the overall excitation light dose, and the distribution of the light dose in time (Magidson and Khodjakov 2013; Tinevez et al. 2012; Logg et al. 2009; Schneckenburger et al. 2012). TRAST imaging requires excitation irradiances high enough to induce population of the transient states. However, using high triplet yield dyes (Spielmann et al. 2014; Mücksch et al. 2015), or dyes for isomerization (Chmyrov et al. 2015), high populations of dark transient states can be reached with lower excitation intensities, and live cell TRAST imaging can be performed with overall excitation light doses almost an order of magnitude lower than estimated maximum tolerable light doses for maintaining cell viability (100 J/cm^2^ at 514 nm irradiation) (Schneckenburger et al. 2012; Wagner et al. 2010). Since TRAST does not rely on fluctuation measurements of single molecules, molecular brightness is not a strongly limiting issue, and low fluorescence signals can be compensated by higher concentrations of fluorescent molecules in the sample, as an alternative to increased excitation irradiances. Thereby, also relatively low brightness compounds, such as autofluorescent species (Hevekerl et al. 2016), can be studied.

### TRAST, examples of biomolecular and cellular information that can be retrieved

**Fig. 4:**
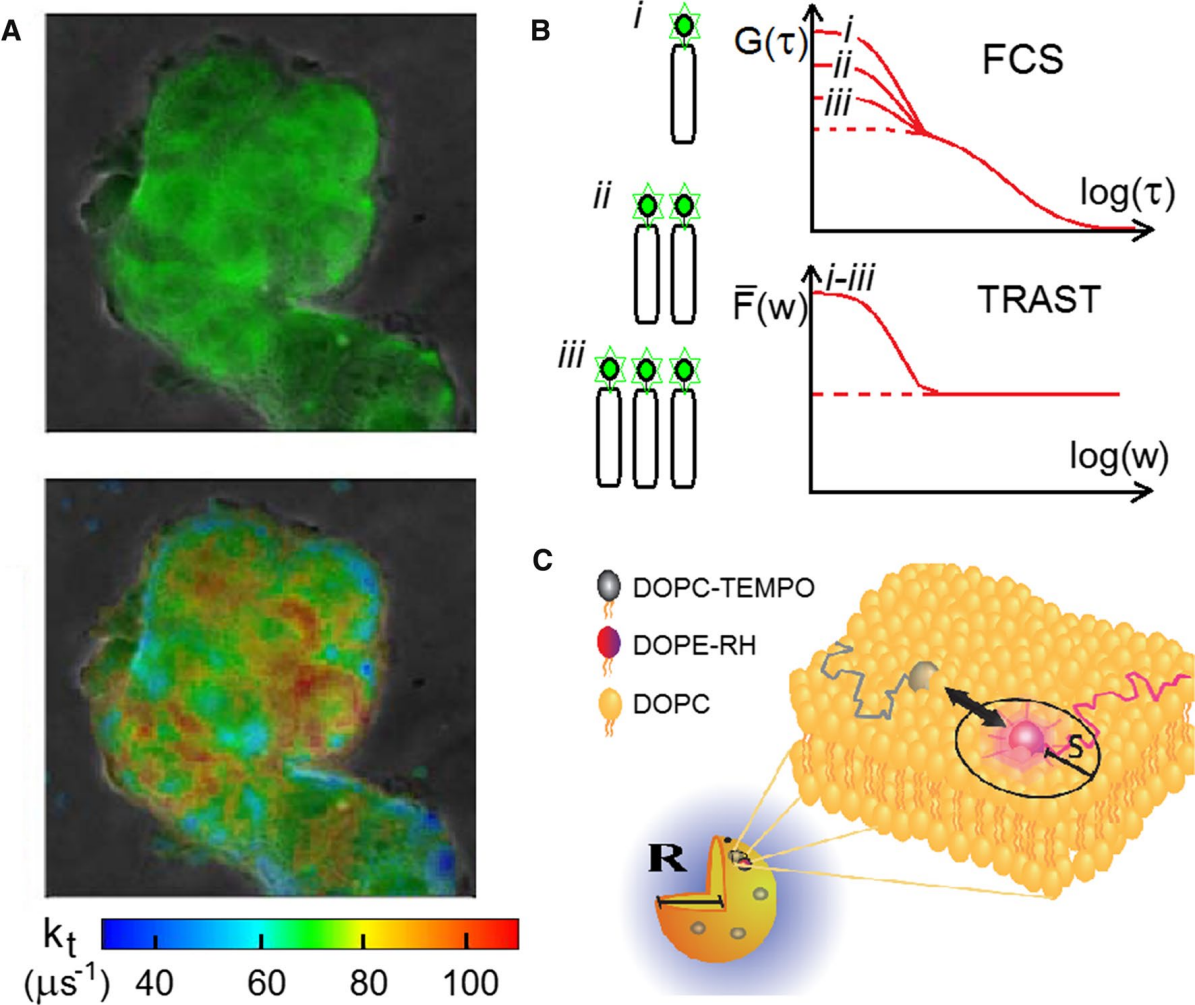
Examples of how TRAST imaging/spectroscopy can be applied. **a** Fluorescence (*top*) and TRAST (*bottom*) images (100 × 100 µm) of cultured cells from a breast cancer cell line (MCF-7), loaded with the fluorophore EosinY. The TRAST image shows the deactivation rate of the *T*_1_ state, *k*_*t*_, obtained from TRAST curves extracted pixel-wise from multiple fluorescence intensity images, recorded with different excitation modulation. **b** Concept for determination of molecular stoichiometry based on FCS and TRAST measurements. Because of the synchronous blinking of the fluorophores in a TRAST experiment, the fluctuation amplitude will not change with several fluorophores on the diffusing units studied. In contrast, in an FCS experiment, the blinking amplitude will decrease with the number of independently blinking emitters on the diffusing units. From the difference in the blinking amplitude in FCS and TRAST, the absolute numbers of fluorophores per diffusing unit can be determined. See main text for further information. **c** Model system used to demonstrate how low-frequency collisional interactions can be monitored via triplet state quenching. Quenching of the triplets states of Lissamine rhodamine B, labeled to DOPE lipids (DOPE-RH), by lipids labeled with the triplet state quencher TEMPO (DOPC-TEMPO) was studied in the membrane of DOPC SUVs. S denotes the quenching radius of interaction (≈ 0.2 nm), and R (≈ 20 nm) the radius of the SUVs. See main text for further details

In Fig. 4, a few examples are given for how TRAST imaging can be applied. Figure 4a shows a fluorescence intensity image (top) and a corresponding image (bottom) of the *T*_1_-to-*S*_0_ rate. This rate is almost completely determined by quenching from dissolved molecular oxygen (Widengren et al. 1995; Spielmann et al. 2014). The latter image is generated by taking consecutive fluorescence intensity images with different excitation pulse trains (pulse durations) applied. The variation of the fluorescence intensity from the excitation modulation (TRAST curves) can then be generated in a pixel-wise manner. By fitting these curves to a photodynamic model of the fluorophores (Fig. 3a), an image of the oxygen-dependent *T*_1_-to-*S*_0_ rate can be obtained. Live cell imaging with this approach allowed the local oxygen concentration within cells to be determined, and characteristic differences in oxygen consumption between normal and cancer cells to be detected (Spielmann et al. 2014; Mücksch et al. 2015). Such differences cannot be resolved by conventional fluorescence imaging parameters, but may be captured by room temperature phosphorescence (RTP). Like TRAST, RTP benefits from long lifetimes, to probe subtle changes in environmental conditions (accessibilities of quenchers, polarities etc.), or to reveal structural and dynamic information of biological macromolecules (Cioni and Strambini 2002). However, while TRAST is based on the readout of a strong fluorescence signal, the RTP signal is weak and susceptible to dynamic quenching by oxygen and trace impurities. Moreover, while TRAST can be based on a broad range of different fluorophores and even auto-fluorescent compounds, specific RTP probes are scarce and cannot easily be loaded into cells (Yu et al. 2017). In contrast to RTP, TRAST is also not limited to imaging of triplet state parameters only. Also other transitions can be imaged, e.g., viscosity-dependent* trans*–*cis* isomerization of lipophilic cyanine dyes, providing images of the microfluidity of cellular membranes (Chmyrov et al. 2015). With a different readout than in established fluorescence-based approaches based on anisotropy, polarity-sensitive dyes, or excimer formation, this TRAST approach offers complementary information and can capture different aspects of membrane microviscosity and fluidity of the cellular membranes.

Figure 4b illustrates a concept to determine molecular stoichiometries, by comparing the fluorescence fluctuations under continuous excitation using FCS, when all the fluorophores on a diffusing unit are blinking independently of each other, with those occurring under square-pulsed excitation using TRAST spectroscopy, when all fluorophores are blinking in a synchronized manner. Thereby, the number of fluorophores per molecule can be determined (Hevekerl and Widengren 2015). In the recorded FCS curves, the fast relaxation amplitude, *B*, of a transient state, is lowered with the number of independently blinking emitters, *N*, on a diffusing unit by *B*/*N*. In contrast, in TRAST measurements the *N* emitters are blinking in synchrony due to the excitation modulation, and the corresponding relaxation amplitude is still *B*. By dividing the TRAST amplitude with the FCS amplitude *N* can thus be obtained directly. The FCS and TRAST measurements can be done consecutively in the same setup. No calibration sample is needed and the approach is independent of experimental conditions and of the specific environment of the molecules under study.

Figure 4c illustrates the principal use of TRAST to monitor low-frequency collisional interactions. In a proof-of-principle study (Strömqvist et al. 2010), we monitored the quenching of long-lived triplet states of Lissamine Rhodamine B dyes, labeled on the head group of lipids. Dye-labeled lipids (DOPE-RH) were placed in the membranes of small unilamellar vesicles (SUVs) with radius *R* ≈ 20 nm. In the SUV membranes we also added varying amounts of lipids (from 0.15% up to 8% of the lipids) labelled with the triplet quencher TEMPO (DOPC-TEMPO). Both the *S*_1_ and *T*_1_ states can be quenched upon interaction between DOPE-RH and DOPC-TEMPO in the SUV membranes (when within a radius of interaction, *S*, of a few Ångström to each other). However, *T*_1_ states have typically at least three orders of magnitude longer lifetimes (µs-ms) than *S*_1_ states (ns), and thus a correspondingly longer time to be influenced by the environment (or quencher collisions). As a result, quenching of *S*_1_, observed as a relative change of the fluorescence lifetime of a few percent, could only be observed for the higher DOPC-TEMPO concentrations, while quenching of *T*_1_ could be observed also for the lowest DOPC-TEMPO concentrations. In other words, interactions between dye-labeled and triplet quencher-labeled molecules can be analyzed via the triplet state population kinetics, at collisional frequencies too low to be reflected as fluorescence intensity or lifetime changes (Strömqvist et al. 2010). This illustrates how transient state monitoring by TRAST (or FCS) can combine the environmental sensitivity of long-lived transient states, with the detection sensitivity of the fluorescence signal, and retrieve information not within reach by conventional fluorescence readouts.

## Conclusions

Molecular relaxation and kinetics measurements have played a key role in reaching a fundamental understanding of a broad range of biological processes. In this review, we have given a historical view of how our research in the development and use of fluorescence correlation spectroscopy and related techniques for such measurements has its roots and has further evolved from the pioneering work of Manfred Eigen and his colleagues and coworkers. Although now 50 years have passed since Eigen, Norrish, and Porter were awarded the Nobel Prize for their achievements in relaxation kinetics measurements, novel molecular fluctuation and relaxation techniques are still under development. This field is thus still not exhausted and will not be in many years to come. Biology is far too intricate. Mother Nature has left many riddles for us to solve, and not unlikely with molecular fluctuation and relaxation measurements required to get hold of many of the central clues.
